# Comparative cognition in three understudied ungulate species: European bison, forest buffalos and giraffes

**DOI:** 10.1186/s12983-021-00417-w

**Published:** 2021-06-22

**Authors:** Alvaro Lopez Caicoya, Federica Amici, Conrad Ensenyat, Montserrat Colell

**Affiliations:** 1 Department of Clinical Psychology and Psychobiology, Faculty of Psychology, University of Barcelona, Barcelona, Spain; 2 Institute of Neurosciences, University of Barcelona, Barcelona, Spain; 3 Behavioral Ecology Research Group, Institute of Biology, University of Leipzig, Leipzig, Germany; 4 Research Group Primate Behavioural Ecology, Department of Human Behavior, Ecology and Culture, Max Planck Institute for Evolutionary Anthropology, Leipzig, Germany; 5 Barcelona Zoo, Barcelona, Spain

**Keywords:** Ungulate, Object permanence, Acoustic cues, Cognition, Bovids, Bison, Buffalo, Giraffe, Test battery, Memory

## Abstract

**Background:**

Comparative cognition has historically focused on a few taxa such as primates, birds or rodents. However, a broader perspective is essential to understand how different selective pressures affect cognition in different taxa, as more recently shown in several studies. Here we present the same battery of cognitive tasks to two understudied ungulate species with different socio-ecological characteristics, European bison (*Bison bonasus*) and forest buffalos (*Syncerus caffer nanus*), and we compare their performance to previous findings in giraffes (*Giraffa camelopardalis*). We presented subjects with an Object permanence task, Memory tasks with 30 and 60 s delays, two inference tasks based on acoustic cues (i.e. Acoustic inference tasks) and a control task to check for the use of olfactory cues (i.e. Olfactory task).

**Results:**

Overall, giraffes outperformed bison and buffalos, and bison outperformed buffalos (that performed at chance level). All species performed better in the Object permanence task than in the Memory tasks and one of the Acoustic inference tasks (which they likely solved by relying on stimulus enhancement). Giraffes performed better than buffalos in the Shake full Acoustic inference task, but worse than bison and buffalos in the Shake empty Acoustic inference task.

**Conclusions:**

In sum, our results are in line with the hypothesis that specific socio-ecological characteristics played a crucial role in the evolution of cognition, and that higher fission-fusion levels and larger dietary breadth are linked to higher cognitive skills. This study shows that ungulates may be an excellent model to test evolutionary hypotheses on the emergence of cognition.

**Supplementary Information:**

The online version contains supplementary material available at 10.1186/s12983-021-00417-w.

## Background

Throughout the history of comparative cognition, there has been a general bias to focus on a few specific species [1, 2], although the inclusion of more diverse taxa can be essential to test specific hypotheses [3, 4]. Such a bias in the selection of study species has often reflected practical considerations (e.g. availability of subjects, maintenance costs) rather than clear research needs. In the 1950 s, for example, few species other than rodents were tested in experimental studies [5]. At the end of the last century, however, the focus has largely shifted on other taxa like primates and corvids [1]. In more recent years, the number of species studied and the research methods used has steadily increased, opening up exciting new possibilities for research in comparative psychology and animal cognition research [6–12]. Despite these recent advances, there is still a long way to go to ensure a fair representation of different taxa in comparative animal cognition research [13].

First of all, the inclusion of species from different taxa can provide important information on the limits of specific evolutionary hypotheses. The fission-fusion hypothesis, for example, predicts that species which frequently split and merge in subgroups of variable size and composition may face enhanced cognitive challenges that might have led to the evolution of specific cognitive skills, like memory, inhibition or analogical skills [14]. However, this hypothesis has mainly been tested in primates, by comparing cognitive performance in a series of species with different degrees of fission-fusion dynamics [15, 16]. Is the fission-fusion hypothesis only valid for primates, or can we extend it to other taxa which also show a similar variation in social dynamics? Including other taxa is therefore a powerful tool to test the limits of specific evolutionary hypotheses and understand whether different selective pressures are at work in different taxa.

Moreover, the study of several species and taxa in cognitive test batteries might provide us with valuable information on how the mind is structured. For example, it has long been debated whether the mind consists of independently evolving modules or if there is a general factor explaining much of the variation in performance observed across different cognitive domains [17, 18]. Several approaches can be used to address this question, and one of these includes comparing the performance of multiple species across different cognitive domains using comparable batteries of cognitive tasks [13]. If some species perform better than others in some domains, but not in all of them, it means that the mind is at least partially modular, with domain-specific cognitive skills probably undergoing different evolutionary pressures in response to specific socio-ecological challenges. Therefore, comparing the performance of several species across several domains allows assessing the extent to which the mind is modular, and also allows indirectly testing which different evolutionary pressures might have selected for specific cognitive skills [19–21].

Ungulates are one of many neglected taxa in comparative cognition, although they are an ideal model to test cognitive skills from a comparative perspective, as demonstrated by several recent studies [22–31]. Although most of these studies have focused on domesticated ungulate species (but see [27, 32]), ungulates also include many non-domesticated species with an impressive variety of socio-ecological characteristics [33], allowing the reliable contrast of different evolutionary hypotheses. Moreover, there are very few studies that have explored the link between cognition and socio-ecological characteristics in ungulates, and all have used neuroanatomical measures as cognitive proxies [33–35]. These studies are promising, and suggest inter-specific differences in cognitive skills: large brains, for instance, are found in species with higher sociality and mixed habitats, while relative neocortex size is usually associated with social (but not ecological) factors [33]. However, neuroanatomical proxies cannot replace direct comparisons of actual cognitive performance [13]. Finally, ungulates are economically crucial for humans and some laboratories have started to study farm animal cognition to improve their welfare, demonstrating how changes in management or facilities can improve animal welfare and economic return [28, 36–44].

In this study, we aimed to test two ungulate species that might prove a valid model to test evolutionary hypotheses on the emergence of cognitive skills in this taxon: European bison (*Bison bonasus*) and forest buffalos (*Syncerus caffer nanus*). These species belong to the same family (Bovidae) and tribe (Bovine), and are therefore phylogenetically very close, although they have very different socio-ecological characteristics. European bison, for example, live in East European forests [45], and although they have physiological adaptations to grazing, they also often browse, so that they are generally considered as mixed feeders [46, 47]. European bison have not been domesticated, and live in herds of about 30 individuals, characterized by high levels of fission-fusion dynamics [48]. In contrast, forest buffalos are a subspecies of the African buffalo that live in dense rainforests in Africa, and feed primarily on grasses [49]. Although high levels of fission-fusion dynamics have been observed in another African subspecies (*Syncerus caffer caffer*), forest buffalo groups are rather cohesive [50], living in small groups of around 15 individuals with stable group size and composition [51, 52]. Moreover, we compared the performance of these two species to giraffes (*Giraffa camelopardalis*), which we had previously tested with the same experimental protocol (see below [27]). Giraffes are browsers with a remarkable dietary breadth [53] that live in open habitats, in fission-fusion societies [27]. By comparing European bison, forest buffalos and giraffes, it is therefore possible to assess whether socio-ecological characteristics (i.e. dietary breadth, fission-fusion dynamics [14, 33, 54]) predict the distribution of certain cognitive skills in ungulates (see below for detailed predictions).

We conducted several tasks in physical cognition on these species, to assess their understanding of objects. Indeed, the ability to segment the world into discrete objects that exist independently of us through space and time is one of the most fundamental conceptual structures, and therefore a widely studied area in comparative cognition [55]. By studying how animals understand objects, for instance, we can gain insight into their ability to deal with several daily physical and social challenges [16]. Object permanence, for example, is a cognitive ability that allows individuals to understand that objects continue to exist even when they are out of sight [56]. This ability is widespread across taxa, and appears to have deep evolutionary roots [56]. In a typical Object permanence task, one of several containers is baited, and once the food is out of view, the subject has to retrieve the food by selecting the container under which the food had been hidden (see [56]). Variations of these tasks include the introduction of a delay between the baiting procedure and the moment in which subjects can retrieve the food, to test subjects’ memory [57]. Another variation of this task provides subjects with an acoustic cue instead of a visual one to locate the baited container [58]. In this task, subjects are presented with two containers, only one having been baited. If subjects understand the causal connection between objects and the noise produced when they move, they should infer that (i) when a container is shaken and produces noise, it likely contains the reward, but (ii) when a container is shaken and produces no noise, the non-shaken container likely contains the reward [22, 58–61].

**Fig. 1:**
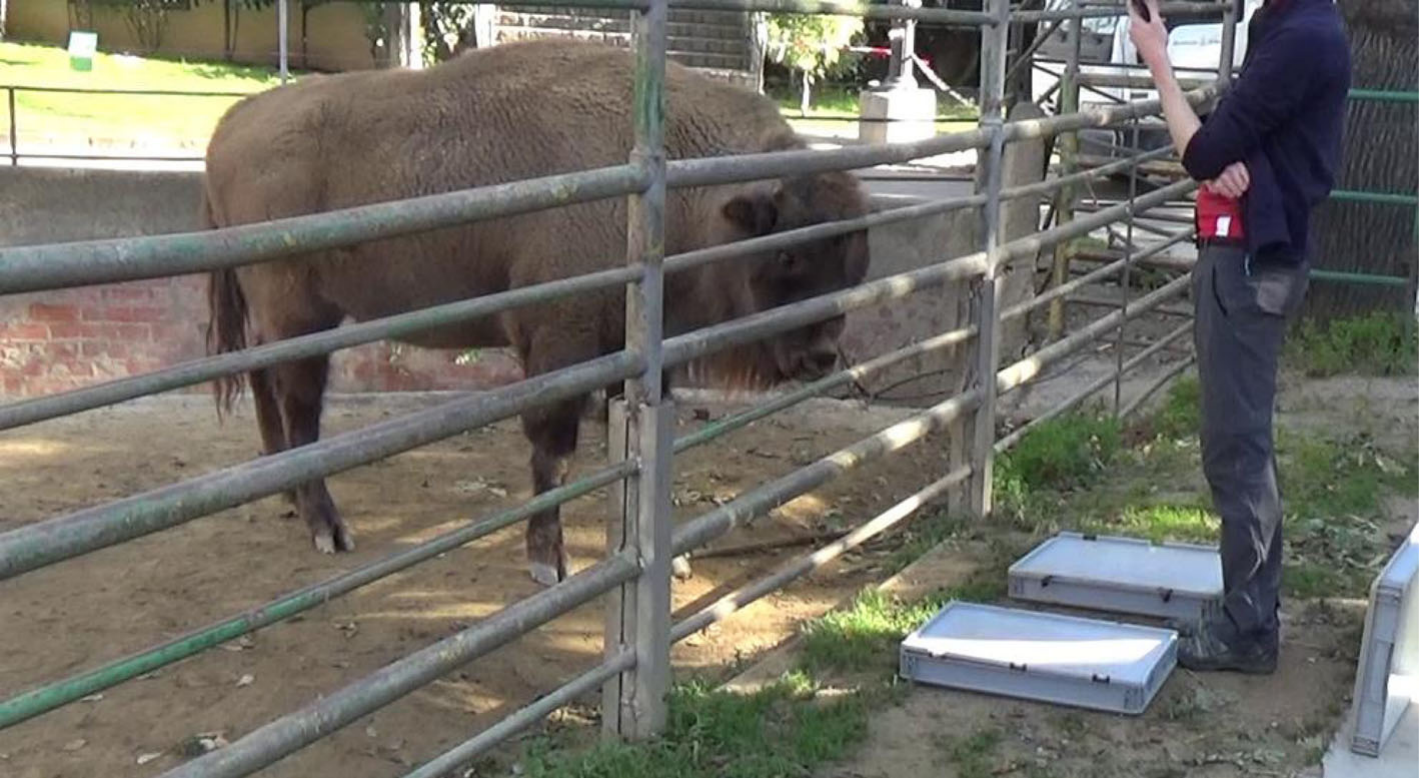
A European bison awaiting in a 30s Memory trial

**Fig. 2:**
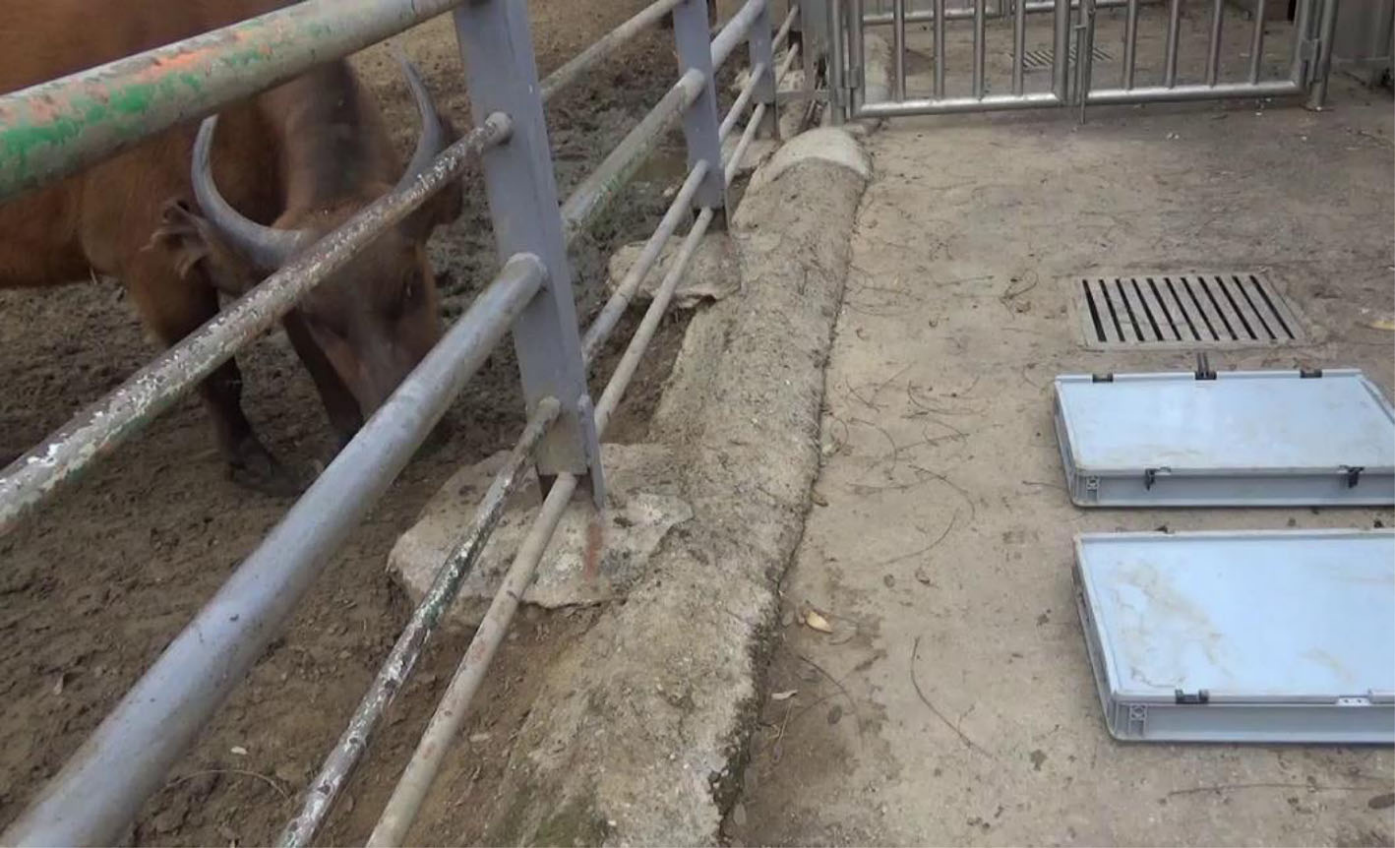
A forest buffalo awaiting in a 30s Memory trial

In this study, we tested European bison and forest buffalos in a series of tasks that were previously conducted in giraffes [27]. These tasks included an Object permanence task, two Memory tasks with 30 and 60 s delays (see Figs. 1 and 2), two Acoustic inference tasks (in which either the container with food rewards or the one without food rewards were shaken), and an Olfactory task (to control that individuals do not use olfactory cues to locate the food). Given that fission-fusion dynamics (e.g. [14]) and dietary breadth (e.g. [7]) have been linked to enhanced cognitive skills (including memory and inferential skills), we predicted that giraffes (forming fission-fusion groups and having a large dietary breadth) would show the best performance in the Memory and Acoustic inference tasks, followed by European bison (which also show a high degree of fission-fusion dynamics but shorter dietary breadth) and lastly by forest buffalos (which live in more cohesive groups and also have shorter dietary breadth). Object permanence, instead, is a rather basic cognitive ability, which appears to emerge relatively early through development and is widespread across animal taxa [56]. Therefore, we predicted that all study species would perform similarly well in the Object permanence task.

## Results

We used a Bayesian approach to assess how performance varied across species depending on the tasks (i.e. Object permanence task, Memory tasks with 30 and 60 s delays, Shake full and Shake empty task, and Olfactory task), whether the position of the food affected performance (i.e. whether individuals showed a side bias, preferentially selecting one side over the other), and whether there was a learning effect (i.e. performance increased across trials). For this reason, we compared a null intercept-only model (M0) to models obtained by adding the following fixed effects: tasks (M1), tasks and species (M2), the 2-way interaction of tasks with species, including their main effects (M3), and the 2-way interactions of tasks with species and food side with species, including their main effects, and trial number (M4; see Table 1).

**Table 1:** List of the models run, ordered with the smallest WAIC (Widely Applicable Information Criteria) and the highest Akaike weight first. For each model, we further present the fixed effects included (main effects were always included in the interactions). Intercept and intercept by subject identity were included in all models. The best model is the first one

Model	Fixed effects included	WAIC	Weight
M4	task*species + side*species + trial	1488.7	1.00
M3	task*species	1538.1	0
M2	task + species	1561.3	0
M1	task	1561.9	0
M0	-	1594.7	0

**Fig. 3:**
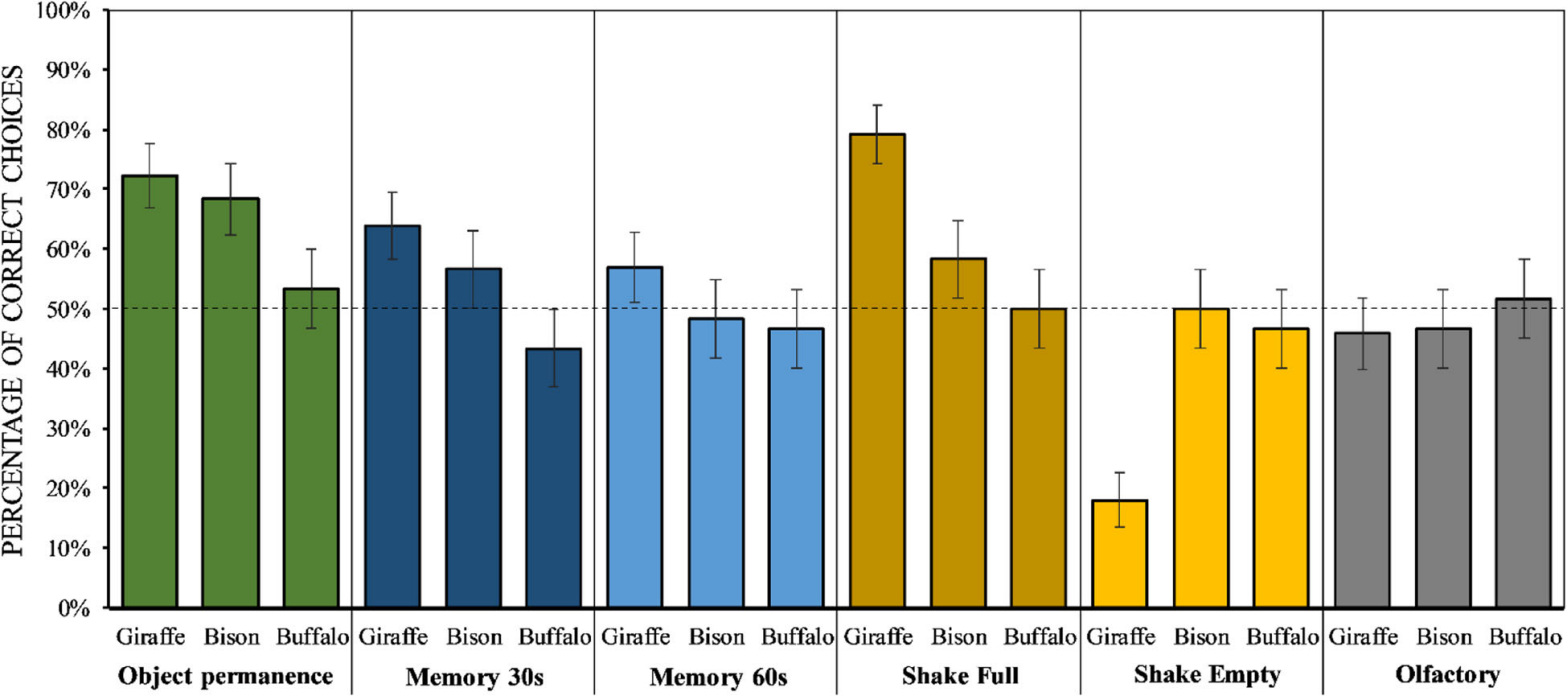
Mean ± SEM of correct choices for each condition and species. The dotted line represents chance level

When comparing models M0 to M4, M4 had the lowest WAIC and the highest model weight (see Table 1). Overall, giraffes were more likely to select the baited container (see Fig. 3), as compared to bison (β = 0.72, 89 % Prediction Interval [PI] = 0.22 to 1.22**)** and buffalos (β = 1.20, 89 % PI = 0.69 to 1.69), while bison were more likely to make the correct choice than buffalos (β = 0.55, 89 % PI = 0.05 to 1.08). As compared to the Object permanence task, performance in all three species was lower in both Memory tasks (with 30 s delay: β = −0.52, 89 % PI = − 1.00 to -0.04; with 60-second delay: β = −0.60, 89 % PI = − 1.06 to -0.12), in the Shake empty task (β = −0.54, 89 % PI = − 1.00 to -0.04) and also in the Olfactory task (β = −0.67, 89 % PI = − 1.14 to -0.19). However, such effect was especially strong for giraffes in the Shake empty task (β = −1.67, 89 % PI = − 2.36 to -1.01). Giraffes were also the only species performing better in the Shake full than in the Object permanence task (β = 0.70, 89 % PI = 0.05 to 1.40). Finally, also the position of the food predicted individuals’ performance, but this effect was weaker in giraffes as compared to both bison (β = -0.89, 89 % PI = -1.34 to -0.44) and buffalos (β = −1.43, 89 % PI = − 1.90 to -0.97), and also weaker in bison than buffalos (β = −0.50, 89 % PI = − 0.96 to -0.03). In contrast, we found no clear effect of trial number, suggesting no increase in performance across trials.

## Discussion

Our study showed important differences in the performance of three ungulate species in a series of tasks on the understanding of objects. In line with our predictions, giraffes showed overall the best performance, followed by European bison and lastly forest buffalos. For all species, performance was highest in the Object permanence task (except for giraffes, that performed better in the Shake full task), which was likely the easiest task, and lower in the two Memory tasks and in the Shake empty task. Importantly, no species relied on olfactory cues to solve the tasks. In contrast to the other species, giraffes performed better in the Object permanence task than in the Shake empty task, but worse than in the Shake full task. Finally, our results showed an effect of food position on individuals’ performance (i.e. side bias), which was strongest in buffalos and intermediate in bison.

Overall, our study provided support to our prediction that the species socio-ecological characteristics predict their cognitive performance, since giraffes showed overall the best performance, followed by bison and lastly by buffalos (see Fig. 3). Giraffes are characterized by large dietary breadth (which has been compared to the dietary breadth of chimpanzees, as both species feed on around 100 different plant species), and high levels of fission-fusion dynamics [62–66]. European bison, in contrast, show high levels of fission-fusion dynamics but short dietary breadth [48], while forest buffalos live in rather cohesive groups and also have short dietary breadth [50]. Therefore, our results would suggest that dietary breadth and/or fission-fusion levels may both contribute to the enhancement of cognitive skills, in line with studies in taxa that have higher encephalization rate (e.g. [15][7]).

However, these results are only preliminary. To confirm them, we would first need to include (i) individuals from more groups, to ensure that our results are independent of the study site, and (ii) larger samples, to better account for inter-individual differences and the possible effect of factors like sex, age or personality [67]. Our bison sample, for instance, only included females, while none of the study species included young individuals. Moreover, we only tested one study group for species (except for giraffes, which were tested in two different zoos). In the future, however, it would be important to include individuals from more groups, as inter-group differences are another important source of variation in the animal kingdom [68]. Therefore, although our results can be easily explained by inter-specific differences in socio-ecological factors, it is not possible to rule out other explanations, especially with our small sample size. Furthermore, we would need to include more species with a wider variety of socio-ecological characteristics (e.g. different predatory pressure, different type of habitat) that might also be linked to inter-specific variation in cognitive skills [33, 69]. Ideally, one should also test a wider range of cognitive skills, as some socio-ecological challenges may be linked to the enhancement of specific cognitive skills. Fission-fusion levels, for instance, have been originally proposed to predict an increase in specific skills, like inhibition and analogical skills, and not to an overall enhancement of cognitive abilities [14]. Testing more cognitive skills would also be essential to understand the extent to which the mind is modular. In this study, all species generally performed better in the Object permanence task than in the other tasks. Therefore, it is not possible to make any inference on intra-specific variation across cognitive domains [17, 18], unless data on more cognitive skills are collected.

Among the study species, buffalos showed the lowest performance, being close to chance levels in all tasks (Fig. 3). Such low performance may be explained by the lack of cognitive skills to solve these tasks, but it may also be due to other reasons, like low motivation or attention during the experiments. To control for that, all species had to pass a habituation phase before being tested (see Methods). This phase ensured that all study subjects (i) were motivated to participate (i.e. they approached the experimenter as soon as he entered in the facilities, and they retrieved all the food during the habituation trials), (ii) were attentive during the experimental procedures (i.e. they observed the experimenter during the baiting procedure) and (iii) understood the basic set-up. Also during the experimental phase, buffalos promptly approached the experimenter when testing started, observed the experimenter during the baiting, and quickly ate the food when choosing the correct side. Furthermore, the average number of sessions (days with experimental trials) required was similar for all the species, subjects required on average 10.1 (± 2.5 SD) sessions to complete the tasks (giraffes: 9.3 ± 2.25 SD; bison: 10.4 ± 2.7 SD; buffalos: 10.8 ± 3 SD). Therefore, we doubt that lack of motivation or lack of attention can explain the inter-specific differences evidenced by our results. Future studies with more individuals should explore whether different set-ups might lead do different performance in this species, as even small procedural changes can importantly affect performance in cognitive tasks [70, 71].

In line with their low performance, buffalos were also more likely to develop side biases, as the position of the food reward had a stronger effect on the choices they made. It is possible that side biases emerged through trials in this species as a response to the difficulty of the tasks, but also that they were the reason why the subjects failed in the tasks. In the first two trials of each task, most subjects showed no side bias (i.e. two subjects selected the right and left container 6 times each, one selected the left container 8 times and the right one 4 times), while two subjects showed a clear initial preference for the left container (which they selected 10/12 times). Through time, however, even the subjects initially showing no side bias developed a preference for one side. Future studies should better assess the tasks triggering the emergence of side biases, and the evolutionary role that these biases play in different species [13, 72].

In the Acoustic inference tasks, giraffes responded differently from bison and buffalos. In particular, giraffes located the baited container in both tasks by reliably choosing the container shaken by the experimenter, regardless of the sound it produced (i.e. likely relying on stimulus enhancement). This turned out in a high number of correct responses in the Shake full task, but in a low number of correct responses in the Shake empty one (see Fig. 3). These results suggest that giraffes might be better than the other study species at attending to humans to locate food. This might depend on the different relationship that giraffes might have with humans at the zoo, although the care given by the keepers to the study subjects was very similar across all study species, and all individuals had undergone the same habituation to the experimenter and the setup. In the future, inter-specific comparisons may benefit from further inclusion of behavioural observations (e.g. to assess personality traits and their effect on cognitive performance) and direct measures of individual reactions to humans, which may also predict cognitive performance [58]. In contrast, these results cannot be explained in terms of giraffes having a better ability to perceive acoustic cues. In the Shake empty task, giraffes performed worse than both other species, suggesting that they relied on the movement of the containers (i.e. stimulus enhancement) rather than on the noise caused by the shaking (i.e. causality), in order to make their choice. If movement (rather than sound) was the criterion that giraffes used to select a container in these tasks, it seems very unlikely that inter-specific differences in the perception of sounds might explain our results. Deeper knowledge about differences in the ability to understand these cues or other human cues could improve the welfare and management of these species in zoos and other facilities.

## Conclusions

Overall this study confirms that ungulates may be an excellent model to test evolutionary hypotheses on the emergence of cognition, complementary to the studies in birds or primates. Despite only including captive individuals, our study revealed important inter-specific differences, suggesting that socio-ecological challenges mainly work in an evolutionary time frame. In the future, it would be necessary to (i) include more ungulate species to confirm these results and contrast more evolutionary hypotheses; (ii) test more individuals to have more robust results and better control for inter-individual variation in performance (e.g. sex, age, rank and personality); and (iii) use larger test batteries to assess a wider range of ungulate cognitive skills (e.g. [73]), and thus contribute to filling the current gaps in our understanding of cognitive evolution.

## Methods

### Aim of the study

We tested three phylogenetically close species in a battery of tasks that measured different cognitive skills (i.e. object permanence, memory and inference skills). We aimed to test whether current evolutionary hypothesis on the link between cognition and socio-ecology (i.e. dietary breadth and fission-fusion) can also explain the distribution of these skills across species with a relatively small encephalization quotient.

### Subjects

We tested five female European bison ranging from 6 to 30 years of age, and two male and three female forest buffalos ranging from 5 to 14 years of age, all housed at the Barcelona zoo, in Spain. Giraffes had already been tested by Caicoya and colleagues [27], and included 6 individuals from 1 to 21 years of age, housed at the zoos of Barcelona, Spain, and Leipzig, Germany (see Table 2). Each study group was housed in enclosures with different size (i.e. giraffes in Barcelona 1.580 m^2^, giraffes in Leipzig 12.260 m^2^, buffalos in Barcelona 835 m^2^ and bison in Barcelona 617 m^2^). In each species, individuals were socially housed with their conspecifics (i.e. social group size for giraffes in Barcelona: N = 3, in Leipzig: N = 7, for buffalos: N = 5, for bison: N = 5). They were all fed on a similar diet based on dry hay, fruit and vegetables. None of the study subjects had previous experience with experimental tasks, and none of them was ever food or water deprived.

**Table 2:** Subjects participating in the study

Species	Name	Sex	Age (years)	Zoo	Rearing history
Forest buffalos (*Syncerus caffer nanus*)	Suza	F	11	Barcelona	Parent
	Joan	M	6	Barcelona	Parent
	Xufa	F	14	Barcelona	Parent
	Canela	F	5	Barcelona	Parent
	Albert	M	14	Barcelona	Parent
European bison (*Bison bonasus*)	Estrella	F	8	Barcelona	Parent
	Verde	F	6	Barcelona	Parent
	Estaca	F	30	Barcelona	Parent
	Espiga	F	14	Barcelona	Parent
	Elipse	F	13	Barcelona	Parent
Giraffes (*Giraffa camelopardalis rothschildi*)	Nuru	F	8	Barcelona	Parent
	Yalinga	F	13	Barcelona	Parent
	Nakuru	M	1	Barcelona	Parent
	Max	M	21	Leipzig	Nursery
	Ashanti	F	16	Leipzig	Mother
	Andrea	F	9	Leipzig	Parent

All subjects were born in captivity.

### Procedures

The experimenter approached the fence of the enclosure from a place only accessible to zoo workers, and waited until one subject approached him. Individuals were always tested in the same area of their enclosure. The first animal approaching the experimenter was tested first, until completion of all the tasks and trials. When more individuals simultaneously approached the experimenter, all but one were made to move in another side of the enclosure by a research assistant using small food baits. In the same way, other group members were prevented from approaching the study subject during testing. Food rewards were always small pieces of carrots (i.e. approximately 5 pieces of 8 g each), which were highly liked food rewards in all study groups. Trials started when the subject’s head was in front of the experimenter, approximately between the two containers.

Before being tested, all individuals and species underwent a habituation phase, to get them used to the experimenter and the set-up. In this phase, we only used one container. The experimenter baited the container out of the subject’s view, turning around to bait it, and then showed the opened container (and its content) to the subject. After 5 s the experimenter closed the lid, waited for 2 s, and pushed the container towards the subject. If the subject touched the container, the experimenter opened the lid and let the subject eat the food. After 4 successful retrievals out of 5 consecutive trials, the subject started the experimental phase. In this way, we ensured to test in the experimental phase only those subjects that were motivated and attentive during the habituation phase, approaching and observing the experimenter during the baiting procedure, and promptly eating the food after having selected the correct container. All the individuals participating in the habituation phase successfully completed it and moved to the next phase.

Upon successful completion of the habituation phase, we started the experimental phase, which consisted of 12 trials for each task. All tasks and trials were administered in a pseudo-randomized order, so that (i) the order of tasks varied across individuals of each species in a similar way, (ii) the right and left container in each task were baited an identical number of times, and (iii) the same side was not baited in more than three consecutive trials. Trials were conducted as long as the subject stayed motivated, and were stopped if the subject failed to approach the experimenter for more than 30 min. In that case, the session was interrupted and testing was resumed on the next possible day, so that the daily number of trials administered varied within and across subjects. We recorded all trials with a video camera (SONY HDR-CX405) fixed on a tripod at one side of the experimenter. The procedures and experimental design used with bison and buffalos exactly matched the ones we had already used with giraffes [27], with the only exception that the opaque containers used for bison and buffalos (i.e. 60 × 40 × 8.4 cm) were larger than the ones used for giraffes (15 × 15 × 3 cm). This change was necessary to ensure that bison and buffalos could retrieve the selected food on their own, as both species are mainly grazers and are not as skilful as giraffes to retrieve food with their tongues.

#### Object permanence task

Out of the subject’s view, the experimenter baited one of the two containers and showed them to the subject, keeping them opened so that their content was visible. The experimenter held both containers approximately 20 cm from each other and around 50 cm from the subject, on the other side of the fence (see Figs. 1 and 2). After 5 s, the experimenter simultaneously closed the lids of both containers, waited for 2 s and then moved both of them toward the subject, who could make a choice. A choice was recorded when the animal touched a box, and the touched box was considered as the one selected by the subject. If the subject touched the correct container, the experimenter opened the lid and let the subject eat the food, while moving the unchosen container out of the subject’s reach. If the subject touched the unbaited container, the experimenter opened its lid and showed its content to the subject, then showed the content of the correct container and removed both. See a video example in Supplementary material “Object Permanence (incorrect trial)”.


**Additional file 1: Video 1.** Object permanence (incorrect trial).

#### Memory task

We used the same procedure as in the Object permanence task. The only difference was the time that elapsed between closing the lid and letting the subject choose. Depending on the task, the time delay was 30 or 60 s, instead of 2 s. See a video example in Supplementary material “Memory 60s (incorrect trial)”.


**Additional file 2: Video 2.** Memory 60s (incorrect trial)

#### Acoustic inference tasks

In these tasks, the experimenter baited one of the two containers out of the subject’s view, so that no visual cues were provided to the subject as to which container was baited. In the Shake full task, the experimenter held both closed containers slightly beyond the subject’s reach, and then shook 3 times the baited container vertically. In this way, the carrots inside the container made a loud noise. After waiting for 2 s, the experimenter simultaneously pushed both containers toward the subject to choose. In the Shake empty task, the procedure was identical, but this time the experimenter shook the empty container, which thus made no sound. If subjects understood that empty containers produce no sound when shaken, they should have inferred that the unshaken container contained food, and preferentially selected it. If subjects instead failed to understand the causal link between the objects and the noise produced when they moved, they might have relied on stimulus enhancement to solve the task (i.e. selecting the shaken container, regardless of whether it produced a sound). See a video example in Supplementary material “Shake Full (correct trial)”.


**Additional file 3: Video 3.** Shake full (correct trial)

#### Olfactory task

We used the same procedure as in the Object permanence task. However, the experimenter never showed the opened containers to the subjects, who could therefore only rely on olfactory cues to locate the food. This task therefore controlled that olfactory cues could not be used to locate the food. See a video example in Supplementary material “Olfactory (incorrect trial)”.


**Additional file 4: Video 4.** Olfactory (incorrect trial)

The experimenter coded the trials on the spot. An observer who was not present during the sessions coded 15 % of all the trials from the video-recordings, which had been randomly selected from the whole pool of trials. Inter-observer reliability was excellent (κ = 0.98, *n* = 162 trials).

### Statistical analyses

We ran multilevel-ordered logit models, always including a varying intercept by subject identity to correct for repeated observations. We included all the administered trials in the data-set, and then assessed variation in correct response. Statistical analyses were run with a Bayesian approach, using the rethinking package [74] in R (version 3.2.3). The Bayesian approach combines prior information about population parameters with sampled data to obtain posterior plausibilities. In all models, we therefore used weakly informative priors to assign the initial plausibilities, and then estimated parameters with RStan (Stan Development Team, 2016). In order to obtain the posterior distribution, we run 3 Hamiltonian Monte Carlo chains in parallel (to reduce autocorrelation within chains), using 10,000 samples, half of which were warm-up. Convergence was suggested by a high number of effective samples (a measure of the extent of autocorrelation of the samples within a chain) and Rhat estimates (measuring convergence of the chains to the target distribution) of 1.00 [74]. We selected models based on the lowest Widely Applicable Information Criteria (WAIC) and the highest Akaike weights. [32]

## Supplementary Information

**Additional file 5.** Dataset.

## Data Availability

All data generated during the study are included in this article as supplementary information file.
